# Can Echocardiographic Findings Predict Falls in Older Persons?

**DOI:** 10.1371/journal.pone.0000654

**Published:** 2007-07-25

**Authors:** Nathalie van der Velde, Bruno H. Ch. Stricker, Jos R. T. C. Roelandt, Folkert J. Ten Cate, Tischa J. M. van der Cammen

**Affiliations:** 1 Department of Internal Medicine, Erasmus University Medical Center, Rotterdam, The Netherlands; 2 Section of Geriatrics, Erasmus University Medical Center, Rotterdam, The Netherlands; 3 Department of Epidemiology & Biostatistics, Erasmus University Medical Center, Rotterdam, The Netherlands; 4 Department of Cardiology, Erasmus University Medical Center, Rotterdam, The Netherlands; Leiden University Medical Center, Netherlands

## Abstract

**Background:**

The European and American guidelines state the need for echocardiography in patients with syncope. 50% of older adults with syncope present with a fall. Nonetheless, up to now no data have been published addressing echocardiographic abnormalities in older fallers.

**Method and Findings:**

In order to determine the association between echocardiographic abnormalities and falls in older adults, we performed a prospective cohort study, in which 215 new consecutive referrals (age 77.4, SD 6.0) of a geriatric outpatient clinic of a Dutch university hospital were included. During the previous year, 139 had experienced a fall. At baseline, all patients underwent routine two-dimensional and Doppler echocardiography. Falls were recorded during a three-month follow-up. Multivariate adjustment for confounders was performed with a Cox proportional hazards model. 55 patients (26%) fell at least once during follow-up. The adjusted hazard ratio of a fall during follow-up was 1.35 (95% CI, 1.08–1.71) for pulmonary hypertension, 1.66 (95% CI, 1.01 to 2.89) for mitral regurgitation, 2.41 (95% CI, 1.32 to 4.37) for tricuspid regurgitation and 1.76 (95% CI, 1.03 to 3.01) for pulmonary regurgitation. For aortic regurgitation the risk of a fall was also increased, but non-significantly (hazard ratio, 1.57 [95% CI, 0.85 to 2.92]). Trend analysis of the severity of the different regurgitations showed a significant relationship for mitral, tricuspid and pulmonary valve regurgitation and pulmonary hypertension.

**Conclusions:**

Echo(Doppler)cardiography can be useful in order to identify risk indicators for falling. Presence of pulmonary hypertension or regurgitation of mitral, tricuspid or pulmonary valves was associated with a higher fall risk. Our study indicates that the diagnostic work-up for falls in older adults might be improved by adding an echo(Doppler)cardiogram in selected groups.

## Introduction

Falls are a major public health hazard in countries with aging populations. Injury is the fifth leading cause of death in older adults, and most of these fatal injuries are related to falls [1]. Falling can be caused by many different risk factors, several of which are cardiovascular [2], [3]. A typical presentation of these cardiovascular causes of falls would be syncopal spells, e.g. a brief loss of consciousness due to loss of blood flow to the brain. However, approximately 50% of older persons do not recall losing consciousness and will therefore present with an unexplained fall instead of syncope [4]. Since the distinction between syncope and falls is so difficult to establish, it is important to address causes of syncope when investigating an older faller.

The main cardiovascular disorders that can cause falls or syncope are orthostatic hypotension, carotid sinus hypersensitivity, vasovagal collapse, cardiac arrhythmias and structural cardiac disease [5], [6]. Although guidelines on syncope state that there is causal evidence for aortic valve stenosis, mitral valve prolapse, outflow-tract obstruction, pulmonary hypertension, and acute myocardial infarction or ischemia, only few studies have reported structural cardiac disease as a causal factor [5]–[7]. According to the American and European guidelines, an echocardiogram is indicated if a patient with syncope is suspected either to have structural heart disease or has an abnormality on the electrocardiogram [5], [6]. Up to now, however, there are no studies addressing the yield of echocardiography in patients presenting with falls. Consequently, we undertook a prospective study in a cohort of geriatric outpatients in which we studied this issue.

## Methods

### Study participants

All new consecutive referrals to the outpatient clinic and the Diagnostic Day Center of the Section of Geriatric Medicine at the Erasmus MC of 65 years or older, with a Mini-Mental State Examination score (MMSE) of 21 points or higher (out of 30 points) [8], [9] and the ability to walk 10 meters without a walking aid, were invited to participate between April 1, 2003 and November 30, 2004. The Medical Ethics Committee of the Erasmus MC approved the study protocol and written informed consent was obtained from all patients.

### Interventions during follow-up

The only intervention performed during the 3 months of follow-up was withdrawal of drugs known to increase fall risk. This consisted of discontinuation or reduction to the lowest possible dose, where possible, in patients with a history of one or more falls during the previous year. The following drugs were considered for withdrawal, i.e. anxiolytics/hypnotics (benzodiazepines and others), neuroleptics (D2 agonists and serotonin dopamine receptor antagonists), antidepressants (tricyclic antidepressants, selective serotonin reuptake inhibitors, serotonin-norepinephrine reuptake inhibitors and monoamine oxidase inhibitors), antihypertensives (diuretics, beta-blockers, alpha-blockers, centrally acting antihypertensives, calcium channel blockers, angiotensin converting enzyme inhibitors and angiotensin receptor blockers), anti-arrhythmics, nitrates and other vasodilators, digoxin, beta-blocker eye drops, analgesics (mainly opioid analgesics), anti-cholinergic drugs, antihistamines, anti-vertigo drugs, and hypoglycemics. The objectives, methods and results of this intervention have been described in detail elsewhere [10].

### Baseline characteristics

Functional status was measured with the Activities of Daily Living scale and the Instrumental Activities of Daily Living scale [11], [12]. We also recorded whether or not study participants used a walking aid. Information on comorbidity was obtained at baseline in an interview with the study participants. This was verified both with the records of the geriatrics department and with information from the general practitioner. All comorbid diagnoses were recorded, including a.o. heart rhythm disorder, ischemic heart disease, cerebrovascular event, hypertension, neurological disorder, depression, COPD, (osteo) arthritis, visual abnormality and total number of comorbid diagnoses. At baseline, several tests were performed. Mobility testing was among others done with the Functional Reach Test, a test for balance. It evaluates the maximal distance that a person can reach forward while maintaining a fixed base of support [13], [14]. Maximal isometric quadriceps femoris muscle strength was measured at both legs with the handheld MicroFET dynamometer [15]. Furthermore, an electrocardiogram was done and a tilt-table test was performed in order to measure orthostatic hypotension, carotid sinus hypersensitivity and vasovagal collapse. Blood pressure was measured performing non-invasive finger arterial pressure measurements as implemented in the Finometer trade mark (Finapres Medical Systems, Amsterdam, The Netherlands).

### Falls history and falls follow-up

A fall was defined as coming to rest unintentionally on the ground or at a lower level with or without losing consciousness, but not if this had been induced by acute medical conditions, such as a stroke, or exogenous factors, such as a traffic accident [16]. For registration of fall incidents during follow-up, respondents were asked to report their falls weekly on a falls calendar and to mail the calendar page at the end of every month. Every participant was called by the first author to check compliance with these calendar pages on a monthly basis. This reporting method is considered the golden standard for research on fall incidents [17], [18]. Furthermore, subjects with a MMSE of 21 or over (our inclusion criterion) have been shown to be able to reliably recall falls [9].

### Echocardiography

Standard echo/Doppler transthoracic examination was performed using a commercially available ultrasound scanner (Sonos 5500, Philips, Best, The Netherlands). Patients were examined in left lateral recumbent position using a standard broadband S4 transducer (2–4 MHz). Echocardiography was performed according to a standard protocol by one trained operator (NV). A second investigator (WF), who was blinded to falls history reviewed all test outcomes and decided on the final scoring.

Standard images were recorded from parasternal long axis, parasternal short axis and apical 4 chamber, apical 2 chamber and apical 3 chamber views. Systolic left ventricular function was scored both qualitatively and quantitatively. Qualitative scoring was performed visually as either good, fair, moderate or poor. For quantitative assessment, ejection fraction and fractional shortening were calculated from left parasternal long axis 2D-guided M mode measurements [19]. For estimation of the ejection fraction we used the simplified method of Quinones *et al* [20]. Standard measurements of the interventricular septum were obtained and a septum thickness of 12 mm or more was considered to be hypertrophic. Valvular functions were assessed using both spectral Doppler and color Doppler. Valvular regurgitation was assessed as mild, moderate, moderately severe or severe on basis of the jet extension (no regurgitation; trivial; 1: mild; 2: moderate; 3: moderately severe: with a long jet; and 4: severe: with a regurgitant jet along the length of either the left ventricle or the right ventricle) [21]. This qualitative assessment is accurate and correlates well with quantitative measures of valvular incompetence [22], [23]. In case of eccentric jets the amount of turbulence was used to estimate the severity of the regurgitation. The degree of valvular stenosis was quantified by continuous wave Doppler velocity measurements using the modified Bernouilli [24]. For velocities through the aortic valve, stenosis was defined as higher than 2.2 m/sec. Systolic pulmonary arterial pressure was estimated when tricuspid regurgitation was detected by continuous wave Doppler echocardiogram, using the peak regurgitant velocity. The pressure gradient between the right ventricle and the right atrium at the time of pulmonary valve opening was calculated by applying the simplified Bernoulli equation (pressure = 4 times velocity2). To this the estimated right artrial pressure was added. Right artrial pressure was estimated at 10 mmHg if the diameter of the vena cava inferior was 1.5 cm or over and during respiration there was a collapse of 50% or over. Right artrial pressure was estimated at 5 mmHg if the diameter of the vena cava inferior was less than 1.5 cm (25). In the absence of tricuspid regurgitation, systolic pulmonary artery pressure was considered normal. In the absence of pulmonary stenosis and right outflow tract obstruction, mild pulmonary hypertension was defined as a systolic pulmonary artery pressure of 35–50 mmHg and severe pulmonary hypertension was defined as a systolic pulmonary artery pressure of 50 mmHg or over [26], [27], [28]. Diastolic function was assessed using Doppler recordings of mitral and pulmonary venous flow (PV) velocities and PV pattern. Impaired relaxation was defined as an E/A ratio<1, atrial reversal flow in the PV>0.4 msec and a PV pattern with S>D. A restrictive pattern was defined as an E/A ratio>1.5, a shortened deceleration time (<160 msec) and a PV pattern with S<D [29].

### Statistical analysis

Baseline differences between the subgroups with and without fall incidents during follow-up were tested using an independent t-test for continuous variables, and a chi-square test for dichotomous variables.

The association between falls during follow-up and cardiac abnormalities was evaluated using a Cox proportional hazards model. First, hazard ratios of events (first fall during follow-up) were computed as estimates of relative risk in a binary fashion (no or trivial abnormalities, versus mild to severe abnormalities). To adjust for possible confounders, cofactors were included one-by-one in the age- and gender-adjusted multivariate model. Cofactors that changed the hazard ratio of a fall incident according to the different echocardiographic abnormalities by more than 5% or that were biologically plausible, and drug withdrawal as defined above, were maintained in the final model. In order to study the association between categories of increasing severity of the valvular regurgitation and risk of a fall, a trend analysis was performed within the Cox proportional hazards model. Thereafter, the variables were treated as categorical variables, in order to get an estimation of the risks for the separate categories of severity of the abnormality. A Kaplan-Meier analysis was performed in order to provide a figure showing the crude survival for the different valvular regurgitations. If numbers were too low in the upper categories, groups were combined in order to get more reliable results, i.e. moderately severe and severe regurgitations were combined to one category: severe. All statistical analyses were performed using SPSS software (version 10.1, SPSS Inc., Chicago, IL, USA).

## Results

During the study period, 350 geriatric outpatients were eligible for inclusion; 132 declined participation, mostly because of the burden of extra visits to the clinic. Patients who refused participation were on average older, used more drugs and had more comorbid diseases. We included 218 patients of whom we were not able to collect data on fall follow-up in 3 subjects: 2 subjects died during follow-up and 1 subject refused further participation during the follow-up period. The remaining 215 subjects were included in the analysis (age 77.4, SD 6.0); Fifty-five patients (26%) fell at least once during follow-up. Baseline characteristics are shown in table 1. Prevalence of echocardiographic abnormalities at baseline is shown in table 2. At baseline, 126 participants used a total of 262 fall-risk-increasing drugs. In 75 participants a total of 91 fall-risk-increasing drugs were withdrawn [10].

**Table 1:** Baseline Characteristics of Study Population.

Baseline characteristics	All	Falls +	Falls −	P-value
	(N = 215)	(n = 56)	(n = 159)	
	n/mean (%/SD)	n/mean (%/SD)	n/mean (%/SD)	
Age, years	77.4 (6.0)	79.1 (6.2)	76.8 (5.9)	0.015
Gender, female	140 (65%)	36 (64%)	104 (65%)	0.88
Referral for falls	109 (51%)	43 (77%)	66 (42%)	0.000
Use of walking aid	86 (40%)	35 (63%)	51 (32%)	0.000
Isometric M. Quadriceps strength, N	208 (91)	192 (99)	213 (87)	0.133
Functional Reach Test, cm	28 (14)	25 (13)	29 (14)	0.028
ADL score	0.52 (1.4)	0.8 (1.6)	0.4 (1.3)	0.070
IADL score	14.2 (3.0)	13.1 (3.5)	14.6 (2.7)	0.005
MMSE score	27.8 (4.9)	27.6 (2.3)	27.8 (5.6)	0.722
Drugs	4.9 (2.8)	5.8 (2.8)	4.6 (2.8)	0.004
Comorbid conditions	4.0 (1.9)	4.8 (1.9)	3.7 (1.8)	0.000
History of angina pectoris	26 (12%)	8 (14%)	18 (12%)	0.602
History of myocardial infarction	24 (11%)	4 (7%)	20 (13%)	0.154
History of atrial fibrillation	22 (10%)	10 (18%)	12 (8%)	0.021
History of other arrhythmia's	15 (7%)	6 (10%)	9 (6%)	0.221
History of cerebrovascular accident	26 (12%)	8 (14%)	18 (12%)	0.602
History of transient ischemic attack	27 (13%)	6 (11%)	21 (13%)	0.669
History of diabetes mellitus	26 (12%)	8 (14%)	18 (12%)	0.602
History of COPD	31 (14%)	11 (20%)	20 (13%)	0.175
History of (osteo)artritis	101 (47%)	34 (61%)	67 (42%)	0.016
History of neurological disorders	18 (9%)	8 (14%)	10 (7%)	0.072
History of depression	28 (13%)	10 (18%)	18 (12%)	0.392
History of visual disorders	54 (25%)	14 (25%)	40 (25%)	0.981
Repolarisation abnormalities, ECG	110 (51%)	28 (50%)	82 (52%)	0.808
Rhythm abnormalities, ECG	128 (60%)	25 (45%)	59 (37%)	0.470
Conduction abnormalities, ECG	135 (63%)	30 (54%)	105 (66%)	0.203
Orthostatic hypotension	130 (61%)	36 (63%)	94 (59%)	0.592
Carotid sinus hypersensitivity	35 (16%)	10 (18%)	25 (16%)	0.735
Vasovagal collapse	15 (7%)	1 (2%)	14 (9%)	0.086

Abbreviations: SD: standard deviation; ADL: activities of daily living; IADL: instrumental activities of daily living; MMSE: Mini-Mental State Examination; COPD: chronic obstructive pulmonary disease; ECG: electrocardiogram.

**Table 2:** Prevalence of Cardiac Abnormalities on Echo(Doppler)cardiography.

Baseline characteristics	All	Falls +	Falls −	P-value
	(N = 215)	(n = 56)	(n = 159)	
	n/mean SD/%	n/mean SD/%	n/mean SD/%	
LV systolic function<good	34 (16%)	11 (20%)	23 (15%)	0.467
LVEF<40%	6 (3%)	1 (2%)	5 (3%)	0.590
Fractional shortening<25%	28 (13%)	5 (9%)	23 (15%)	0.308
Diastole: impaired relaxation	160 (74%)	37 (66%)	123 (77%)	0.031
Diastole: restrictive pattern	5 (3%)	4 (8%)	1 (1%)	0.031
Aortic valve stenosis (≥mild)	20 (9%)	4 (7%)	16 (10%)	0.532
Aortic valve regurgitation (≥mild)	55 (25%)	16 (29%)	38 (24%)	0.459
Mitral valve regurgitation (≥mild)	70 (32%)	24 (43%)	46 (29%)	0.064
Tricuspid regurgitation (≥mild)	97 (45%)	37 (67%)	60 (37%)	0.000
Pulmonary regurgitation (≥mild)	73 (33%)	26 (47%)	46 (29%)	0.014
Pulmonary hypertension (≥mild)	47 (22%)	16 (29%)	31 (19%)	0.579
Tricuspid reg. velocity>2,5 m/s	44 (20%)	16 (29%)	28 (18%)	0.452
LV diameter systole (mm)	30 (8)	30 (8)	29 (7)	0.360
LV diameter diastole (mm)	47 (7)	48 (9)	47 (7)	0.380
LV hypertrophy (>12 mm)	72 (33%)	21 (36%)	53 (33%)	0.725

Abbreviations: LV, left ventricular; LVEF, left ventricular ejection fraction; reg., regurgitation.

**Figure 1:**
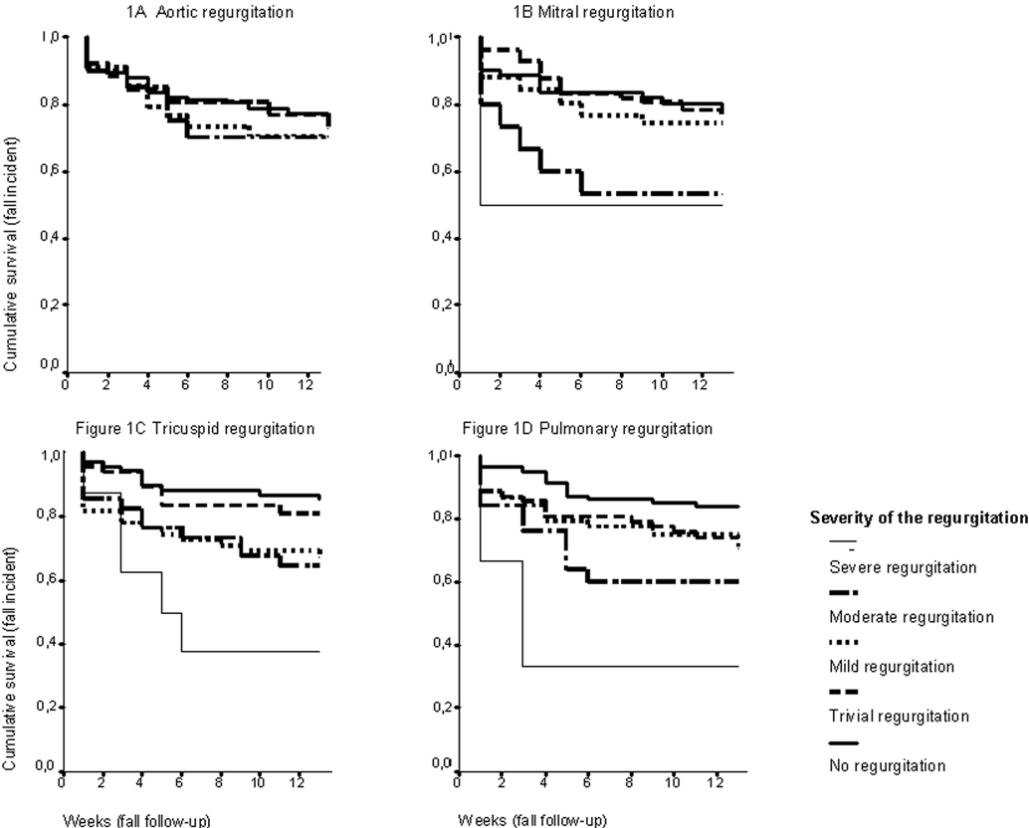
Kaplan-Meier Curve of Valve Regurgitation (Aortic, Mitral, Tricuspid and Pulmonary) According to Falls Incidence, Divided for Severity of the Regurgitation.

First, the hazard ratio of fall incidents according to echocardiographic abnormalities was calculated in a binary fashion: relative risk of falls in patients with at least a mild echocardiographic abnormality (regurgitation, stenosis, pulmonary hypertension, left ventricular ejection fraction, fractional shortening, left ventricular hypertension) compared to patients without or with only a trivial abnormality. For the associations, the following potential cofactors were considered and tested for possible confounding: age, gender, referral for falls, cognitive function (MMSE-score), use of fall-risk-increasing drugs, withdrawal of fall-risk-increasing drugs, use of cardiovascular drugs, withdrawal of cardiovascular drugs, comorbid conditions: total number and separately (heart rhythm disorder, ischemic heart disease, cerebrovascular event, hypertension, neurological disorder, depression, COPD, (osteo)arthritis, visual abnormality), use of a walking aid, ADL and IADL function, presence of abnormalities on the electrocardiogram, presence of orthostatic hypotension, carotid sinus hypersensitivity, vasovagal collapse, presence of other abnormalities on the echocardiography besides the one under consideration. Our final analysis included age, gender, MMSE-score, number of co-morbid conditions and use and withdrawal of fall-risk-increasing drugs as cofactors. This resulted in a higher risk that was statistically significant for mitral valve regurgitation, tricuspid valve regurgitation and pulmonary valve regurgitation (table 3). A statistically significant relationship between increased pulmonary hypertension, as measured with tricuspid regurgitation velocity and and systolic pulmonary arterial pressure, and falls during follow-up was also found, which was significant after adjustment for the cofactors mentioned above, including presence of valvular regurgitations. No significant correlation with falls was found for aortic valve stenosis, aortic valve regurgitation, left ventricular function<40%, fractional shortening<25%, or left ventricular hypertrophy. However, the number of participants small, especially for poor left ventricular function, therefore giving rise to uncertain results (table 3). In a second analysis, the association between categories of increasing severity of valvular regurgitation and pulmonary hypertension and the risk of a fall was studied (figure 1). With the exception of aortic valve regurgitation, the relative risk increased with increasing severity of the regurgitation and there was a significant trend for mitral, tricuspid and pulmonary regurgitation (table 4). Trend analysis for pulmonary hypertension was also significant.

**Table 3:** Hazard Ratio of a Fall according to Echocardiographic Abnormalities (N = 215).

Echocardiographic finding	HR, Model 1 (95% (CI)	P-value	HR, model 2 (95% CI)	P-value
Aortic valve stenosis (≥mild stenosis)	0.60 (0.22–1.68)	0.330	0.63 (0.22–1.76)	0.375
Aortic valve regurgitation (≥mild regurgitation)	1.15 (0.63–2.08)	0.650	1.57 (0.85–2.92)	0.154
Mitral valve regurgitation (≥mild regurgitation)	1.64 (1.08–2.84)*	0.038	1.66 (1.01–2.89)*	0.049
Tricuspid valve regurgitation (≥mild regurgitation)	2.51 (1.39–4.53)*	0.002	2.41 (1.32–4.37)*	0.004
Pulmonary valve regurgitation (≥mild regurgitation)	1.81 (1.06–3.08)*	0.030	1.76 (1.03–3.01)*	0.040
Tricuspid valve regurgitation velocity (linear)	2.85 (1.20–6.76)*	0.018	2.69 (1.09–6.65)*	0.032
Pulmonary hypertension (≥mild PH)	1.60 (1.02–2.50)*	0.039	1.35 (1.08–1.71)*	0.023
LV hypertrophy (septum>12 mm)	1.47 (0.77–2.82)	0.136	1.78 (0.87–3.64)	0.148
LVEF (<40%)	1.61 (0.22–11.65)	0.624	1.51 (0.21–10.97)	0.670
Fractional shortening (<25%)	1.98 (0.71–5.51)	0.335	2.18 (0.78–6.11)	0.255

Model 1: adjusted for age, gender.

Model 2: adjusted for age, gender, number of comorbid conditions, Mini-Mental State Examination, drug intervention during follow-up.

Abbreviations: HR, hazard ratio; CI, confidence interval; PH, pulmonary hypertension; LV, left ventricular; LVEF, left ventricular ejection fraction.

* P<0.05.

**Table 4:** Trend Analysis of Hazard Ratio of a Fall and Aortic, Mitral, Tricuspid and Pulmonary Valve Regurgitation (N = 215).

	N	HR (95% CI)	P-value
Trend aortic valve regurgitation (linear)		1.16 (0.90–1.50)	0.248
No aortic valve regurgitation	134	1.00 (reference)	
Aortic valve regurgitation (trivial)	26	1.00 (0.42–2.43)	0.994
Aortic valve regurgitation (mild)	35	1.80 (0.85–3.80)	0.123
Aortic valve regurgitation (moderate)	20	1.30 (0.52–3.24)	0.518
Aortic valve regurgitation (severe)	0	- -	-
Trend mitral regurgitation (linear)		1.35 (1.07–1.72)*	0.013
No mitral valve regurgitation	61	1.00 (reference)	
Mitral valve regurgitation (trivial)	83	1.32 (0.64–2.74)	0.453
Mitral valve regurgitation (mild)	51	1.70 (0.77–3.73)	0.188
Mitral valve regurgitation (moderate)	16	2.28 (0.88–5.93)	0.091
Mitral valve regurgitation (severe)	4	3.79 (1.05–13.76)*	0.043
Trend tricuspid valve regurgitation (linear)		1.46 (1.17–1.83)*	0.001
No tricuspid valve regurgitation	69	1.00 (reference)	
Tricuspid valve regurgitation (trivial)	48	1.72 (0.68–4.36)	0.255
Tricuspid valve regurgitation (mild)	55	2.94 (1.30–6.65)*	0.009
Tricuspid valve regurgitation (moderate)	35	2.91 (1.19–7.09)*	0.019
Tricuspid valve regurgitation (severe)	8	5.13 (1.71–15.36)*	0.003
Trend pulmonary valve regurgitation (linear)		1.36 (1.08–1.71)*	0.009
No pulmonary valve regurgitation	80	1.00 (reference)	
Pulmonary valve regurgitation (trivial)	63	1.89 (0.91–3.94)	0.090
Pulmonary valve regurgitation (mild)	44	2.17 (1.02–4.65)*	0.045
Pulmonary valve regurgitation (moderate)	25	2.52 (1.09–5.87)*	0.031
Pulmonary valve regurgitation (severe)	3	4.08 (0.87–19.28)	0.076
PAPs (linear)		1.05 (1.01–1.09)*	0.017
PAPs<35 mmHg (reference)	169	1.00 (reference)	
PAPs 35–50 mmHg (mild PH)	39	1.14 (0.53–2.45)	0.737
PAPs>50 mmHg (severe PH)	8	3.31 (1.18–9.29)*	0.023

Hazard ratio adjusted for age, gender, number of comorbid conditions, Mini-Mental State Examination, drug intervention during follow-up.

Explanation severity regurgitation: mild, 1+; moderate, 2+; severe, 3+ & 4+.

Abbreviations: RR, relative risk; CI, confidence interval; PAPs, pulmonary arterial systolic pressure; PH, pulmonary hypertension.

* P<0.05.

## Discussion

In our study, several echo(Doppler)cardiographic abnormalities were risk indicators for falls. First of all, risk of falls was increased if regurgitation of the mitral, tricuspid or pulmonary valve was present. The level of risk increased according to the severity of the regurgitation. Valvular disease is known to cause syncope, the main evidence being for aortic valve stenosis and mitral valve prolapse [5], [6], [30], [31]. The guidelines on syncope state that structural heart disease can cause syncope when circulatory demands outweigh the impaired ability of the heart to increase its output. Valvular regurgitation will impair peak cardiac output, and if the circulatory demands cannot be met, this will result in a shortage of cerebral perfusion. In older patients, this can either present as a fall or as a syncopal spell. Although the recent guidelines pertain to syncope [5], [6], our results suggest that these guidelines may also apply to older fallers. Another possible explanation besides the possible causal chain mentioned above, is the fact that cardiac abnormalities might act as predictors for frailty, since in the Cardiovascular Health Study, frailty has been shown to predict falls in older persons and in this same cohort it has also been shown that subclinical cardiovascular disease was associated with frailty [32], [33]. However, no significant relationship between frailty and left ventricular ejection fraction or frailty and mitral valve abnormalities was found, making it unlikely that these abnormalities acted as markers for frailty. They did not provide data on tricuspid, aortic or pulmonary valve abnormalities. Furthermore, in our analysis, cofactors associated with frailty, i.e. sort and number of comorbidities, age, cognitive function, use of a walking device and ADL and IADL function, did not act as confounding factors.

An increased fall risk was also found for high tricuspid regurgitation velocity and high pulmonary systolic pressure, which was used as a proxy for pulmonary hypertension. Our finding is therefore in line with earlier publications showing a high percentage of pulmonary hypertension in syncope patients [5], [6]. The number of patients with pulmonary hypertension (defined as a pulmonary systolic pressure ≥35 mm Hg) was high in our population, i.e. 22%. This is in line with the findings of a study by Dokainish et al, showing a prevalence of 23% in patients aged at least 90 years old and further normal echocardiography [28]. This is an interesting finding because it may not reflect physiological aging but it possibly indicates a high number of non-identified morbidity in this age group. This would be in line with the high percentage of undetected pulmonary embolisms in this age group, as was found in a recent study of older persons presenting with acute respiratory failure to the emergency department [34].

Although further research will be needed to confirm our findings and establish the clinical usefulness of echocardiographic screening in older fallers, it is interesting to contemplate on the possible clinical consequences and effects, e.g. treatment and a possible ensuing decrease in fall risk. First of all we want to emphasize the fact that most fall incidents in older persons are of multifactorial origin and therefore treatment of valvular abnormalities will need to be part of a multifactorial intervention. Assuming that there is indeed a causal relationship between falls and valvular abnormalities, care of older fallers may be improved by implementing echocardiography in the clinical work-up for older fallers. However, further research will be needed first to determine the clinical added value of including echocardiography to the falls assessment. Furthermore, the available treatments need some consideration in order to judge the potential decrease in fall risk. Surgical repair might be feasible for a subgroup of geriatric patients. Still, it is likely that the majority will not benefit from such a drastic procedure, partly because their cardiac abnormalities are not severe enough to engage in a surgical procedure and partly because in a fair amount of geriatric patients comorbidity is significant, giving rise to an unacceptably high per-operative risk. Therefore, in most patients diligent review of possible precipitants including tailoring of drug treatments will be the intervention of choice. In case of pulmonary hypertension optimal treatment depends of course on the underlying disorder, but could consist of anticoagulants, optimizing treatment for COPD and other lung disorders including oxygen therapy, a trial period with calcium channel blockers (may be either beneficial or detrimental), digoxin, or advanced therapy including prostanoids, endothelian receptor agonists and PDE5 inhibitors. For patients with mitral regurgitation and atrial fibrillation, cardioversion and antiarrhythmic drugs are recommended. Furthermore, in case of mitral regurgitation a reduction of preload with beta-blockers, calcium channel blockers or diuretics can be advantageous, in severe cases vasodilator therapy including ACE inhibitors and angiotensin II receptor blockers may be of benefit. However, data regarding the beneficial effect of long-term vasodilator therapy are conflicting. ACE inhibitors or angiotensin II receptor blockers may be of use in case of comorbity (hypertension, diabetes or left ventricular dysfunction). Besides surgery, cardiac resynchronization may be of use. Reduction of systolic blood pressure is the primary goal in mitral valve prolapse and rheumatic valve disorders, using either beta-blockers, diuretics, hydrazaline or calcium channel blockers. For pulmonary regurgitation and tricuspid regurgitation evidence regarding optimal conservative treatment is limited, but consists of treatment of pulmonary hypertension if present and lowering of the afterload [35], [36]. Since vigilant conservative treatment of valvular abnormalities and pulmonary hypertension includes the use of known fall-risk-increasing drugs, we think that is of utmost importance to carefully weigh the benefits and dangers of treatment, and for this, certainty of presence of the cardiac abnormality is needed. Therefore, assuming that there is indeed a causal relationship between these cardiac abnormalities and fall incidents, in our opinion, echocardiographic assessment will be needed if treatment is considered.

Well-known causes for syncope are aortic valve stenosis and left ventricular outflow-tract obstructions. Contrary to our expectations, an increased fall risk was not found for these conditions. This is most probably caused by the fact that only 20 cases of aortic valve stenosis were present in our cohort, the majority being mild (n = 17) and probably only moderate to severe aortic valve stenosis will result in deficient cardiac output in demanding situations.

Our study has some potential limitations. First, in a subgroup of our cohort drugs known to increase fall risk were withdrawn. This intervention was completed after the first month and reduced fall incidence for the intervention group in the second and third month of follow-up [10]. However, adjustment for the intervention did not change our results (table 3 and 4), thus making it unlikely that this intervention acted as a confounder in the association between echocardiographic abnormalities and falling. A second potential limitation of our study is the substantial number of patients who refused participation. On average this group was older and frailer, which might have given rise to a generalizability problem. It seems unlikely that selection bias explains the positive association between valvular regurgitation and fall risk. On the contrary, we expect that the risk would have been even higher in the non-participants because the old and frail have less effective cardiovascular coping mechanisms.

Furthermore, we have assessed 9 predefined possible associations, giving rise to the possibility of random error due to multiple testing. However, since the associations in question were all defined a-priori on the bases of a clinical hypothesis and a significant result due to chance (random error) occurs only one in twenty times (using p<0.05) we do not think it likely that our findings were caused by chance.

In conclusion, this study has shown that in our cohort of geriatric outpatients there was an increased risk of falls in patients with regurgitations or mitral, tricuspid or pulmonary valves and in patients with pulmonary hypertension. To our knowledge, this is the first study addressing the association between echocardiographic abnormalities and falls in older persons. Our study demonstrates that the incidence of cardiac abnormalities is high and that there is an association between cardiac abnormalities and fall incidents. Therefore, we think that a two-dimensional echo(Doppler)cardiogram might be useful in the diagnostic work-up of selected groups of older fallers. Echocardiographic screening of older fallers can be important for two reasons. First, it can provide a diagnosis in a fair number of cases, and second, it can help select those patients who need careful tailoring of their (drug) treatments. Since this is the first study addressing this topic, more studies will be needed to determine the need for and the yield of standard echocardiography in older fallers.
